# Japanese Encephalitis Virus in Australia: From Known Known to Known Unknown

**DOI:** 10.3390/tropicalmed4010038

**Published:** 2019-02-20

**Authors:** Andrew F. van den Hurk, Alyssa T. Pyke, John S. Mackenzie, Sonja Hall-Mendelin, Scott A. Ritchie

**Affiliations:** 1 Public Health Virology, Forensic and Scientific Services, Department of Health, Queensland Government, PO Box 594, Archerfield, QLD 4108, Australia; Alyssa.Pyke@health.qld.gov.au (A.T.P.); Sonja.Hall-Mendelin@health.qld.gov.au (S.H.-M.); 2 Faculty of Medical Sciences, Curtin University, and Division of Microbiology and Infectious Diseases, PathWest, Locked Bag2009, Nedlands, WA 6909, Australia; J.Mackenzie@curtin.edu.au; 3 College of Public Health, Medical and Veterinary Sciences, and Australian Institute of Tropical Health and Medicine, James Cook University, PO Box 6811, Cairns, QLD 4870, Australia; scott.ritchie@jcu.edu.au

**Keywords:** Japanese encephalitis virus, zoonosis, mosquito, transmission, Australia

## Abstract

Japanese encephalitis virus (JEV) is a major cause of neurological disease in Asia. It is a zoonotic flavivirus transmitted between water birds and/or pigs by *Culex* mosquitoes; humans are dead-end hosts. In 1995, JEV emerged for the first time in northern Australia causing an unprecedented outbreak in the Torres Strait. In this article, we revisit the history of JEV in Australia and describe investigations of JEV transmission cycles in the Australian context. Public health responses to the incipient outbreak included vaccination and sentinel pig surveillance programs. Virus isolation and vector competence experiments incriminated *Culex annulirostris* as the likely regional vector. The role this species plays in transmission cycles depends on the availability of domestic pigs as a blood source. Experimental evidence suggests that native animals are relatively poor amplifying hosts of JEV. The persistence and predominantly annual virus activity between 1995 and 2005 suggested that JEV had become endemic in the Torres Strait. However, active surveillance was discontinued at the end of 2005, so the status of JEV in northern Australia is unknown. Novel mosquito-based surveillance systems provide a means to investigate whether JEV still occurs in the Torres Strait or is no longer a risk to Australia.

## 1. Introduction

Japanese encephalitis virus (JEV) is a single-strand, positive-sense RNA virus of the genus *Flavivirus*, family *Flaviviridae*. The virus is responsible for approximately 68,000 clinical cases annually and is the leading cause of encephalitis in a number of countries in Southeast Asia, the Indian sub-continent and the Indonesian archipelago [1]. Predominantly asymptomatic, less than 1% of human infections result in clinical disease which can range broadly in severity from a mild febrile illness to acute meningomyeloencephalitis. Of symptomatic cases, 20–30% are fatal, and among the survivors, approximately 30–50% will have ongoing neurological sequelae. 

Prevalent in tropical and subtropical parts of Asia and the Pacific rim [2], JEV exists in a zoonotic transmission cycle between ardeid wading birds, such as herons and egrets, and *Culex* mosquitoes, particularly, *Culex tritaeniorhynchus* and *Cx. vishnui* which utilize rice fields for larval development [3]. Domestic pigs are important amplifying hosts, due to rates of infection of 90–100%, development of viremia levels sufficient to infect mosquitoes and constant annual turnover leading to a continual supply of immunologically naïve pigs as susceptible hosts. Recent experiments have demonstrated that JEV can be transmitted directly between pigs via oronasal secretions further enhancing the status of pigs as amplifying hosts [4]. Although the epidemiological significance of this finding needs to be definitively established, it suggests that virus transmission can potentially occur in the absence of suitable mosquito vectors. Humans and horses can develop fatal disease, but they are considered dead end hosts of JEV because they do not produce adequate viral levels required to infect mosquitoes. Thus, JEV is considered an important zoonotic pathogen and a concerted One Health approach is required for sustained disease suppression [5].

**Figure 1:**
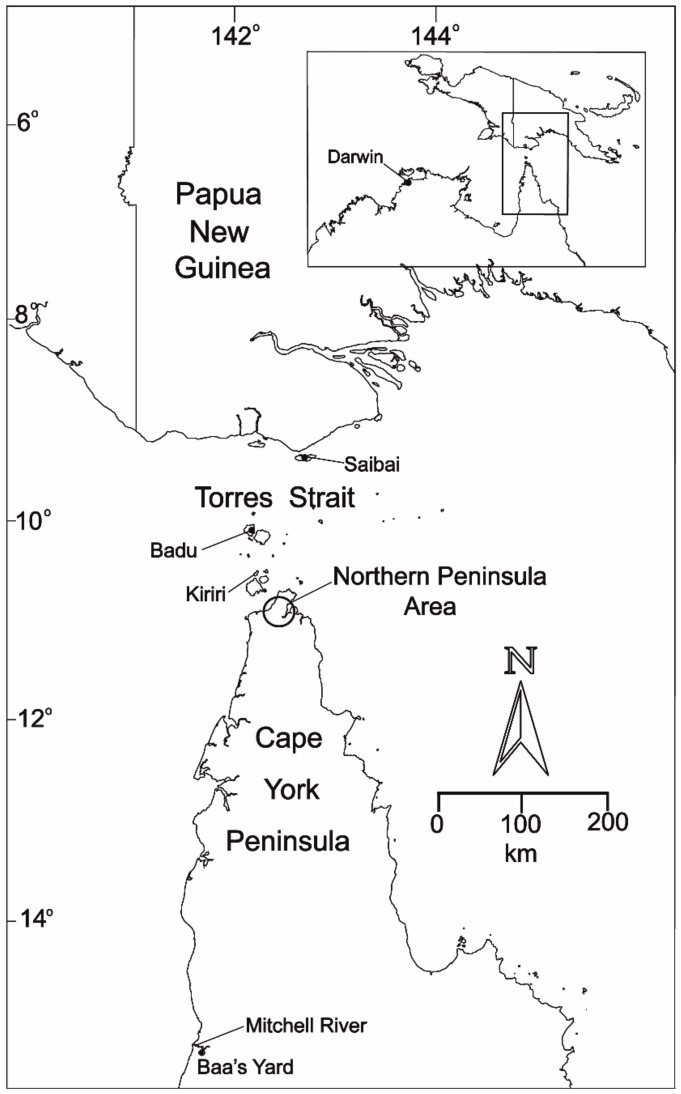
Map of Northern Australia and southern Papua New Guinea showing locations mentioned in the text. The Northern Peninsula Area includes the communities of Bamaga, Injinoo, New Mapoon, Seisia, and Umagico.

In Australia, JEV is mostly viewed as an issue for travelers to endemic regions and occasional overseas acquired cases are reported [6,7,8]. However, in 1995, JEV was first recognized in natural transmission cycles in northern Australia when a widespread outbreak occurred on the islands of the Torres Strait, the body of water that separates Cape York Peninsula and the New Guinea landmass (Figure 1). Three human cases, two of which were fatal, occurred on the island of Badu. This event was unprecedented, as Murray Valley encephalitis virus (MVEV) and West Nile virus Kunjin subtype (WNV_KUN_) were considered the only encephalitogenic flaviviruses southeast of Wallacea, the region that separates the Asian and Australasian zoogeographical regions. In the current paper, we revisit the epidemiology of JEV in the Australasian region and summarize research conducted to elucidate the factors that led to its emergence and apparent disappearance.

## 2. The Emergence of JEV in Northern Australia

In March 1995, Public Health authorities were notified of three cases of encephalitis on Badu in the Torres Strait [9]. Given previous MVEV activity in northern Australia, it was initially suspected that this virus was the etiological agent. However, virus isolation from serum samples from two asymptomatic residents of the island and mosquitoes yielded JEV isolates. Several other residents and domestic pigs were also found to be JEV seropositive by enzyme linked immunosorbent assay (ELISA) and hemagglutination inhibition (HAI) assay. Whilst it is known that flavivirus cross-reactivity can obscure serological findings and complicate result interpretation, a proportion of these serum samples also demonstrated specific neutralizing antibodies at notably higher titers to JEV than to MVEV or WNV_KUN_, providing further definitive evidence that JEV had caused the outbreak [9]. Virus genotyping revealed that the Badu 1995 human and mosquito isolates clustered within genotype II [9]. *Culex annulirostris* was the only mosquito that yielded JEV at a carriage rate of 2.97 per 1000 mosquitoes, indicating that this was the potential mosquito vector [10]. Subsequent, broader serosurveys of humans and pigs revealed that the outbreak was widespread across the Torres Strait, although Badu appeared to have conditions conducive to epizootic JEV transmission. This included an immunologically naïve human population and a large immunologically naïve domestic pig population, with numerous pigpens located close to houses. There were also widespread productive larval habitats of *Cx. annulirostris*, created by poorly maintained drains, damaged septic systems, and groundwater sites which had become nutrient rich due to feces from horses that had been introduced to the island in the year preceding the outbreak [11]. Emergency vector control strategies included treatment of larval sites with the insect growth regulator, *s*-methoprene, and thermal fogging of adults with the pyrethroid, bioresmethrin.

Following this incipient outbreak, several strategies were employed to limit JEV transmission, both on Badu and on other islands. A vaccination program using the formalin inactivated mouse brain-derived vaccine (Biken Institute, Japan) commenced in December 1995 and by March 1996, 93% of residents of the outer islands who commenced the schedule had received at least 2 doses [12]. To detect further JEV activity, a sentinel pig program was established on 4 islands of the Torres Strait, as well as proximal to mainland Australian communities on the tip of Cape York Peninsula [13]. To reduce the availability of larval habitats, maintenance and drainage works were initiated on Badu, although the swampy ground present over much of the island limited the impact of this strategy on adult mosquito populations [11]. In 1996 and 1997, JEV activity, as evidenced by seroconversions of sentinel pigs, appeared restricted to the northernmost island of Saibai.

The unexpected emergence of JEV in the Australasian region prompted investigations of the origins of the virus and potential mechanisms of introduction. Between 1996 and 1998, almost 400,000 mosquitoes were processed from the Western Province of Papua New Guinea (PNG) yielding 3 isolates [14]. Furthermore, there was evidence of human infection in PNG, as demonstrated by JEV-specific antibodies in sera collected as far back as 1989 [15] and by clinical cases of encephalitis (J Oakley and S. Flew, unpublished data cited by [14]. Thus, it appeared that the New Guinea landmass was the source of the incursions. Furthermore, backtrack simulations by Ritchie and Rochester [16] suggested that wind-borne mosquitoes carried by low pressure systems from New Guinea could have been the mechanism of virus introduction.

In 1998, widespread JEV activity again occurred in the Torres Strait and, for the first time, on the Australian mainland [17]. There were two clinical human cases recognized serologically during this outbreak, with the first being in an unvaccinated child on Badu and the second being a fisherman at the mouth of the Mitchell River on western Cape York Peninsula. Sentinel pigs on many outer islands seroconverted to the virus, whilst seroconversion of pigs on Kiriri Island signaled the first evidence of transmission occurring on the inner Torres Strait islands. On the mainland, sentinel pigs on the Northern Peninsula Area (NPA) and at Baa’s Yard near the Mitchell River, seroconverted to JEV, and the virus was isolated from 3 pigs at Seisia on the NPA. Collections on Badu yielded 42 isolates of JEV from 31,898 mosquitoes, with all but one coming from *Cx. sitiens* subgroup mosquitoes (primarily *Cx. annulirostris*); the other isolate was from *Aedes vigilax* [18]. In contrast, no JEV was detected in 35,235 mosquitoes processed from the Australian mainland [19]. Nucleotide sequence analysis and molecular genotyping of mosquito and pig 1998 isolates revealed that they also belonged to genotype II. A high nucleotide identity was also demonstrated between the sequences of the 1998 and 1995 Torres Strait viruses, and to the Australian mainland and PNG sequences, highly suggesting that the origin of JEV incursions into Northern Australia may have been PNG [17,18]. Following the 1998 outbreak, and with a view to reducing contact and transmission between pigs, mosquitoes, and humans, domestic pigs were relocated from proximal to houses to communal pig pens >2 km away from the community. 

The magnitude of the 1998 outbreak of JEV in the Torres Strait and Cape York Peninsula was unprecedented in both spatial scale and intensity. JEV activity was recorded from southern PNG, across most of the Torres Strait and south to western Cape York Peninsula [14,17], suggesting a unique and extreme event. The outbreak appeared to represent the convergence of high populations of *Culex* mosquitoes and widespread JEV transmission in southern PNG, coupled with a strong tropical cyclone in the Gulf of Carpentaria that may have transported JEV infected mosquitoes from PNG into the Torres Strait and deep into Cape York Peninsula. Late 1997 to early 1998 featured a strong *El-Nino* event that caused severe drought in the Western Province of PNG [20]. Normally flooded wetlands may have been reduced to stagnant pools of highly organic water favorable for the production of *Cx. sitiens* subgroup mosquitoes. Indeed, mosquito collections in February 1998 in Western Province were very high, with many traps catching over 10,000 mosquitoes per night [16], from which JEV was isolated [14]. This event was also coupled with the occurrence of Tropical Cyclone Sid in the western Gulf of Carpentaria in late December 1997. The large wind field of this category 2 cyclone was potentially sufficient to transport mosquitoes from southern New Guinea to west central Cape York Peninsula, where JEV activity occurred at the mouth of the Mitchell River [16]. The coincidental occurrence of two extreme events, drought induced JEV transmission in southern PNG and a cyclone in the Gulf of Carpentaria that could transport the mosquitoes from New Guinea landmass deep into the Cape York Peninsula, make a repeat of this event unlikely. 

After no recognized activity in 1999, JEV reappeared in the Torres Strait in 2000. Although virus was not isolated from 7652 *Cx. annulirostris* collected from Badu, a single JEV isolate was obtained from 84 *Cx. gelidus* mosquitoes [21]. Collections from Saibai Island also yielded an isolate, albeit from *Cx. sitiens* subgroup mosquitoes [22]. JEV isolates were also obtained from the acute sera of three pigs on Badu. Interestingly, molecular genotyping of the two mosquito and pig sera 2000 isolates demonstrated they belonged to genotype I and did not cluster with the previous Australian 1995 and 1998 genotype II viruses. Importantly, this demonstrated the introduction of a new JEV genotype into Australia and highlighted the continued risk and vulnerability of the region to further JEV incursions [22,23]. 

Between 2001 and 2005, sentinel pigs and/or deployment of a newly developed remote mosquito trapping system were effective in detection of JEV on Badu Island every year [24]. In 2004, the virus was again detected on mainland Australia, when pigs located on the NPA seroconverted to JEV and a single isolate was obtained from a pool of *Cx. sitiens* subgroup mosquitoes collected from the Bamaga rubbish dump [25]. This was the first time that JEV had been isolated from mosquitoes collected from the Australian mainland. Molecular phylogenetic analysis revealed that the virus clustered with a 2004 Badu pig isolate, and 2000 mosquito and pig sequences in genotype I. As no further evidence of genotype II in the region had been demonstrated since 1998, these findings suggested this genotype may have been subsequently replaced by genotype I. 

The sentinel pig program and remote mosquito trapping trials were discontinued in the Torres Strait at the end of the 2005, whilst the sentinel pigs were removed from the NPA in 2011. However, given the continual risk of re-emergence, in 2012, mosquito-based surveillance was re-deployed, albeit using a different system (refer to Section 3.3) in the NPA by the Northern Australia Quarantine Service. Despite multiple detections of MVEV and WNV_KUN_ in the NPA traps which were most notable in 2015 [26], there has been no evidence of recent JEV activity (T. Kerlin and K. Rickart, unpublished data). Traps were also run on Badu, but only during the 2012–2013 wet season. No JEV was detected during this period of deployment. Thus, the status of JEV in the Torres Strait since 2005 is unknown. 

## 3. Elucidating the Ecology of JEV Transmission Cycles in Australia

### 3.1. Vertebrate Host Studies

Numerous vertebrate species have been investigated as amplifying hosts of JEV in endemic regions, although ardeid birds and pigs are considered the most important [2]. When JEV appeared in northern Australia, it was feared that the large populations of feral pigs and wading birds on the mainland would provide an abundance of amplifying hosts to allow the virus to become established in natural transmission cycles. An unknown quantity and continuing concern is the role that other vertebrates, particularly native species, could play in these transmission cycles. Unfortunately, laboratory based vertebrate studies are very complex, requiring high level biocontainment, which restricts them to a limited number of laboratories in Australia. Thus, there has only been limited experimentation on the course of JEV infection in Australian vertebrates. In experiments conducted well before JEV emerged in Australia, the Nankeen Night Heron, *Nycticorax caledonicus*, was shown to produce viremia levels that could potentially infect recipient mosquitoes [27]. Later, the response of marsupials to JEV infection was investigated at the Australian Animal Health Laboratories (AAHL). It was shown that eastern grey kangaroos, agile wallabies and tammar wallabies either did not develop detectable viremia or were only capable of producing viremia levels below the threshold required to infect questing mosquitoes (PW Daniels, D Middleton, D Boyle, K Newberry, D Williams, R Lunt, unpublished data cited by Mackenzie et al. [28]). In contrast, possums produced a higher viremia when compared to the macropods tested. The only other native species examined as a potential amplifying host in laboratory-based experiments was the black flying fox, *Pteropus alecto* [29]. Only 1 of 15 flying foxes produced a detectable viremia which was sufficient to infect recipient mosquitoes. Interestingly, 3 other flying foxes were able to infect recipient mosquitoes, even though they did not produce a viremia that was detectable using a highly sensitive real-time reverse transcriptase PCR. Despite exhibiting low infection rates following experimental exposure, flying foxes could still play a role in the ecology of JEV in Northern Australia, as they roost in camps containing 1000s of individuals, are prevalent on a number of islands of the Torres Strait and are known to migrate from the New Guinea landmass. 

The importance of pigs as amplifying hosts of JEV and the existence of large, abundant feral populations across Northern Australia prompted pig infection studies with a regional context. Of particular interest, was whether prior exposure to endemic MVEV or WNV_KUN_ viruses affected pig susceptibility to JEV infection and how this may impact on their immune responses. In experiments performed at AAHL, JEV was readily detected in pigs following primary JEV infection, but not in pigs previously infected with MVEV or WNV_KUN_ that were later challenged with JEV [30]. Coupled with suppressed JEV viremia levels, elevation of existing cross-reactive JEV neutralizing antibodies were further demonstrated in these pigs. Notably, these findings suggest that prior exposure to MVEV or WNV_KUN_ may elicit protective immunity against JEV in pigs. Together with the suppression of viremia levels, this indicates that pre-immune pigs may not be effective amplifying hosts and therefore are unlikely to play a major role in JEV transmission.

### 3.2. Incrimination of Mosquito Vectors

In the majority of regions where JEV is known to circulate, *Cx. tritaeniorhynchus* and *Cx. vishnui* are the key mosquito vectors, however, these species, do not occur in Northern Australia. Based on its role as the primary vector of MVEV and WNV_KUN_ [31], it was suspected that an alternate species, *Cx. annulirostris*, was the primary vector during the original Torres Strait 1995 outbreak. This hypothesis was further supported by the fact that this was the only species from which JEV isolates were recovered during this initial outbreak and to date, has been the species yielding the most isolates obtained in Northern Australia. Subsequent laboratory-based vector competence experiments using genotype II JEV isolated from Badu Island in 1998 confirmed the status of *Cx. annulirostris* as the likely primary vector in Australia [32]. Interestingly, Hemmerter et al. [33] demonstrated that *Cx. annulirostris* contains at least 5 mitochondrial cytochrome oxidase I lineages, with some having a wide distribution in the Australasian regions, whilst others appeared more restricted geographically. It was revealed that three of these lineages occurred in southern PNG, the Torres Strait and Cape York Peninsula. The authors hypothesized that these lineages may vary in their vector competence for JEV, thus potentially explaining the southern limits of the virus on the Australian mainland. Phenotypic evidence to corroborate this hypothesis was provided by Johnson et al. [34] who showed that the dominant mainland Australian lineage of *Cx. annulirostris* was a relatively poor laboratory vector of the genotype I JEV. 

Studies of other species which yielded isolates demonstrated that *Cx. gelidus* was a highly efficient laboratory vector [34] whereas *Ae. vigilax* had a comparatively low transmission rate [32]. Although other species, such as *Cx. sitiens* and *Cx. quinquefasciatus* were efficient laboratory vectors [32] and have been implicated as secondary vectors in SE Asia, their status as vectors in Northern Australia remains largely unknown. Finally, electrophoretic analysis of collections of *Cx. annulirostris* that yielded JEV revealed that the closely related and morphologically similar *Cx. palpalis* was present, sometimes at high levels [35]. Thus, this species could also be considered a potential JEV vector. Similar to the situation in endemic locations, the evidence from virus detection in field collected mosquitoes and vector competence experiments incriminates members of the genus *Culex* as the primary vectors of JEV in Northern Australia. 

### 3.3. The Influence of Mosquito Host Feeding Patterns on JEV Transmission in Northern Australia

The propensity for the mosquito to feed on the vertebrate host is critical to its role as a virus vector. Analysis of host feeding patterns of *Cx. annulirostris* from numerous locations in northern Australia revealed that, for the most part, pigs and birds accounted for only a small percentage of positive blood meals [36,37]. Instead, most of the blood meals obtained by *Cx. annulirostris* originated from marsupials, particularly the Agile Wallaby, *Macropus agilis* [37,38]. As mentioned previously, experiments conducted at AAHL had previously shown that Agile wallabies produced only low-level viremia. Thus, predilection for *Cx. annulirostris* to feed on wallabies may have dampened transmission, particularly on the mainland, by diverting host seeking mosquitoes away from pigs. Interestingly, there are no wallabies on Badu, so this possible dampening effect would not have impacted transmission dynamics on the island. 

The only locations where significant feeding on pigs was recorded in northern Australia was from locations adjacent to domestic pigs or where feral pigs congregated, such as rubbish dumps. In endemic areas in Southeast Asia, intense JEV transmission is usually driven by pig feeding rates >30%. Thus, it was not surprising when analysis of *Cx. annulirostris* host feeding patterns during periods of recognized JEV transmission also revealed relatively high porcine feeding rates, as high as 80% [36,37]. The proportion of *Cx. annulirostris* feeding on pigs was significantly reduced when the domestic pigs were relocated from the Badu community to a communal piggery some 2.5 km away [21]. However, this did not appear to eliminate virus transmission close to human habitation, as infected mosquitoes were subsequently collected within the community [39], although it may have diminished the potential for transmission. It was suggested that domestic pigs needed to be moved further away to be out of the flight range of *Cx. annulirostris*, which can be as much as 12 km [40].

### 3.4. Development of Mosquito-Based JEV Surveillance Systems

Undoubtedly, the sentinel pig surveillance program played a considerable role in providing evidence of JEV activity and, in some cases, seroconversion in herds preceded human cases [17]. However, the use of sentinel pigs for detection of viral activity has several limitations affecting their continued deployment, particularly in remote areas [24]. Firstly, the fact that pigs are a key amplifying host of the virus is an obvious risk which may exacerbate and contribute to ongoing transmission. Secondly, sentinel animal programs can be highly expensive to establish and maintain, impacting on local resources and resulting in a major financial burden to biosecurity and public health authorities. Efficient running and effectiveness of sentinel animal programs may also be affected by labor-intensive bleeding and collection procedures which can lead to occupational health and safety issues and, if delayed, can greatly affect downstream result interpretation and disease management strategies. The inherent difficulty in distinguishing JEV from MVEV and WNV_KUN_ infections in serological assays due to cross-reacting antibodies may also obscure accurate laboratory interpretations and require further testing by highly specialized reference laboratories [30].

A surveillance system involving detection of viral RNA in mosquitoes collected in continuously run mosquito traps has been developed as an alternative to using sentinel pigs. The original iterations of this mosquito-based system involved processing all mosquitoes collected in solar or propane-powered traps [24]. However, these traps had the capacity to collect >150,000 mosquitoes in a week, so diagnostic capacity was overwhelmed. To circumvent the need to process hundreds of pools, a system was developed that takes advantage of the sugar feeding behavior of mosquitoes [41]. In this system, mosquitoes are collected in CO_2_-baited traps, where they can feed on honey-soaked nucleic acid preservation cards, which are submitted for detection of viral RNA [42]. A number of modifications have been made to the trapping system, resulting in the current trap design, the sentinel mosquito arbovirus capture kit (SMACK) which does not require electricity and maximizes survivability of collected mosquitoes, thus increasing the likelihood of multiple feedings on the cards [26,43].

The sugar-based arbovirus system has been trialled in several locations around Australia and has detected a number of arboviruses, including MVEV and WNV_KUN_, the alphaviruses, Ross River and Barmah Forest viruses, and the bunyavirus Gan Gan [26,42,44,45]. Detection of WNV_KUN_ in traps deployed near Darwin, Northern Territory, without concurrent detection in sentinel chickens demonstrated that the system is potentially more sensitive than sentinel animals in some instances [45]. Furthermore, if enough viral RNA is expectorated on the cards, it can provide a template for nucleotide gene sequencing in phylogenetic studies. The sugar-based arbovirus system using SMACK traps is now deployed operationally at 12 remote locations in Queensland, including communities in the NPA. Although JEV has not been detected in cards removed from field-deployed traps, results from laboratory-based studies showed that this virus could readily be detected in saliva expectorated by sugar-feeding mosquitoes [41]. This indicates that the sugar-based system has direct utility for JEV surveillance. 

In the current sugar-based arbovirus system, the small amounts of virus expectorated on the nucleic acid preservation cards means that some samples deemed positive are at the limit of detection in molecular assays [45]. To increase the sensitivity of the sugar-based surveillance system for arbovirus detection, investigations are currently underway into the utility of mosquito excreta as an alternative sample type to saliva [46]. Early results have demonstrated that both WNV_KUN_ and dengue viruses can be detected in excreta at a higher rate than it is detected in the saliva, and possibly represents the greater volume of liquid excreted by mosquitoes (1.5 µL) compared with the volume of saliva expectorated (4.7 nL) [46,47].

## 4. Conclusions

To date, the detection of JEV in mosquitoes collected in a mosquito trap on Badu in March 2005 signified the final time that virus activity was unequivocally detected in Northern Australia. Overall, the virus was detected in 10 out of 11 years between 1995 and 2005 indicating that JEV had either become established in enzootic cycles in the Torres Strait or was re-introduced during the period almost annually when conditions were suitable. 

Despite the status of the JEV in the Torres Strait being largely unknown, the ongoing vaccination program has likely limited the number of human cases. The vaccines currently utilized in the Torres Strait are the live attenuated, recombinant vaccine (IMOJEV) and the inactivated, African green monkey kidney (Vero) cell culture-derived vaccine (JEspect) [48]. Vaccination is recommended for residents of the outer islands and non-residents who spend a cumulative total of 30 days in the Torres Strait during the wet season (December to May) and, as such, is offered as part of an immunization program in risk areas.

The lack of evidence of JEV activity on Badu since 2005 likely represents limited surveillance rather than natural disappearance of the virus from the region. Human infection with JEV resulting in clinical disease is rigorously investigated and is defined as clinical evidence of non-encephalitic and encephalitic disease coupled with definitive laboratory testing [49]. However, the majority of JEV infections are asymptomatic and the only human virus isolates obtained from the initial 1995 outbreak were from two asymptomatic patients and these were only recovered after wider, retrospective sampling and surveillance of Torres Strait island residents was performed. Thus, some other form of active surveillance could potentially provide evidence as to whether the virus is still circulating in the Torres Strait. 

When JEV first emerged in northern Australia it was initially feared that the virus would proliferate in mosquito–pig–bird cycles and become established on the mainland [50] similar to events involving establishment of WNV in bird-mosquito cycles in the United States [51]. Despite these predictions, viral activity appears to have remained restricted to the Torres Strait, with the occasional incursion onto Cape York Peninsula. There is no evidence to suggest that the virus has become established on the mainland, let alone reaching endemic status in any other areas of the country. Several ecological reasons for this apparent lack of establishment have been proposed and include: (a) competition between the endemic flaviviruses, MVEV and WNV_KUN_, with JEV for susceptible vertebrate hosts; (b) host feeding patterns of *Cx. annulirostris* whereby they feed on hosts other than pigs, that cannot amplify JEV; and (c) different lineages of *Cx. annulirostris* which vary in their vector competence for the different genotypes of JEV [3]. Alternatively, the lack of detection on the mainland could represent the limited geographical area covered by the current sugar-based surveillance program. Whilst a vaccination program is in place for Torres Strait island residents, immunologically naïve populations exist on the mainland. Thus, it would be prudent to continue the current JEV surveillance program on Cape York Peninsula, and consider expanding its geographical scope with increased sensitivity to provide future early warning and enhanced public health prevention of disease. 

The investigations presented in the current paper are, in effect, examples of One Health in action. Indeed, a One Health approach has been successfully used to understand JEV transmission and to provide tools to combat epidemics [5], and it has been suggested that the employment of One Health strategies, particularly those concerned with improving coordination and collaboration across different disciplines and jurisdictions, are essential to planning and initiating interventions to mitigate risk and in improving prevention and control of mosquito-borne arboviruses [52].

## References

[1] Campbell G.L., Hills S.L., Fischer M., Jacobson J.A., Hoke C.H., Hombach J.M., Marfin A.A., Solomon T., Tsai T.F., Tsu V.D. (2011). Estimated global incidence of Japanese encephalitis: A systematic review. Bull. World Health Organ..

[2] Mackenzie J.S., Williams D.T., Smith D.W. (2006). Japanese encephalitis virus: The geographic distribution, incidence, and spread of a virus with a propensity to emerge in new areas. Perspect. Med. Virol..

[3] Van den Hurk A.F., Ritchie S.A., Mackenzie J.S. (2009). Ecology and geographical expansion of Japanese encephalitis virus. Ann. Rev. Entomol..

[4] Ricklin M.E., García-Nicolás O., Brechbühl D., Python S., Zumkehr B., Nougairede A., Charrel R.N., Posthaus H., Oevermann A., Summerfield A. (2016). Vector-free transmission and persistence of Japanese encephalitis virus in pigs. Nat. Commun..

[5] Impoinvil D.E., Baylis M., Solomon T. (2013). Japanese encephalitis: On the One Health agenda. Curr. Top. Microbiol. Immunol..

[6] Fleming K. (1975). Japanese encephalitis in an Australian soldier returned from Vietnam. Med. J. Aust..

[7] Hanson J.P., Taylor C.T., Richards A.R., Smith I.L., Boutlis C.S. (2004). Japanese encephalitis acquired near Port Moresby: Implications for residents and travellers to Papua New Guinea. Med. J. Aust..

[8] Macdonald W.B.G., Tink A.R., Ouvrier R.A., Menser M.A., de Silva L.M., Naim H., Hawkes R.A. (1989). Japanese encephalitis after a two-week holiday in Bali. Med. J. Aust..

[9] Hanna J.N., Ritchie S.A., Phillips D.A., Shield J., Bailey M.C., Mackenzie J.S., Poidinger M., McCall B.J., Mills P.J. (1996). An outbreak of Japanese encephalitis in the Torres Strait, Australia, 1995. Med. J. Aust..

[10] Ritchie S.A., Phillips D., Broom A., Mackenzie J., Poidinger M., van den Hurk A. (1997). Isolation of Japanese encephalitis virus from *Culex annulirostris* in Australia. Am. J. Trop. Med. Hyg..

[11] Ritchie S.A., van den Hurk A.F., Shield J. (1997). The 1995 Japanese encephalitis outbreak: Why Badu?. Arbovirus Res. Aust..

[12] Hanna J., Barnett D., Ewald D. (1996). Vaccination against Japanese encephalitis in the Torres Strait. Comm. Dis. Intell..

[13] Shield J., Hanna J., Phillips D. (1996). Reappearance of the Japanese encephalitis virus in the Torres Strait, 1996. Comm. Dis. Intell..

[14] Johansen C.A., van den Hurk A.F., Ritchie S.A., Zborowski P., Paru R., Bockari M.J., Drew A.C., Khromykh T.I., Mackenzie J.S. (2000). Isolation of Japanese encephalitis virus from mosquitoes (Diptera: Culicidae) collected in the Western Province of Papua New Guinea, 1997–1998. Am. J. Trop. Med. Hyg..

[15] Johansen C., Ritchie S., Hurk A.v.d., Bockarie M., Hanna J., Phillips D., Melrose W., Poidinger M., Scherret J., Hall R. (1997). The Search for Japanese encephalitis virus in the Western Province of Papua New Guinea, 1996. Arbovirus Res. Aust..

[16] Ritchie S.A., Rochester W. (2001). Wind-blown mosquitoes and introduction of Japanese encephalitis into Australia. Emerg. Infect. Dis..

[17] Hanna J.N., Ritchie S.A., Phillips D.A., Lee J.M., Hills S., van den Hurk A.F., Pyke A., Johansen C.A., Mackenzie J.S. (1999). Japanese encephalitis in north Queensland, Australia, 1998. Med. J. Aust..

[18] Johansen C.A., van den Hurk A.F., Pyke A.T., Zborowski P., Phillips D.A., Mackenzie J.S., Ritchie J.S. (2001). Entomological Investigations of an outbreak of Japanese encephalaitis virus in the Torres Strait, Australia, in 1998. J. Med. Entomol..

[19] Van den Hurk A.F., Johansen C.A., Zborowski P., Phillips D.A., Pyke A.T., Mackenzie J.S., Ritchie S.A. (2001). Flaviviruses isolated from mosquitoes collected during the first outbreak of Japanese encephalitis virus on Cape York Peninsula, Australia. Am. J. Trop. Med. Hyg..

[20] Barr J. (1999). Drought Assessment: The 1997-98 El Nino Drought in Papua New Guinea and the Solomon Islands. Aust. J. Emerg. Manag..

[21] Van den Hurk A.F., Nisbet D.J., Johansen C.A., Foley P.N., Ritchie S.A., Mackenzie J.S. (2001). Japanese encephalitis on Badu Island, Australia: The first isolation of Japanese encephalitis virus from *Culex gelidus* in the Australasian region and the role of mosquito host-feeding patterns in virus transmission cycles. Trans. Royal. Soc. Trop. Med. Hyg..

[22] Johansen C.A., Nisbet D.J., Foley P.N., van den Hurk A.F., Hall R.A., Mackenzie J.S., Ritchie S.A. (2004). Flavivirus isolations from mosquitoes collected from Saibai Island in the Torres Strait, Australia, during an incursion of Japanese encephalitis virus. Med. Vet. Entomol..

[23] Pyke A.T., Williams D.T., Nisbet D.J., van den Hurk A.F., Taylor C.T., Johansen C.A., Macdonald J., Hall R.A., Simmons R.J., Mason R.J.V. (2001). The appearance of a second genotype of Japanese encephalitis virus in the Australasian region. Am. J. Trop. Med. Hyg..

[24] Ritchie S.A., van den Hurk A.F., Zborowski P., Kerlin T.J., Banks D., Walker J.A., Lee J.M., Montgomery B.L., Smith G.A., Pyke A.T. (2007). Operational trials of remote mosquito trap systems for Japanese encephalitis virus surveillance in the Torres Strait, Australia. Vector Borne Zoonotic Dis..

[25] Van den Hurk A.F., Montgomery B.L., Northill J.A., Smith I.L., Zborowski P., Ritchie S.A., Mackenzie J.S., Smith G.A. (2006). The first isolation of Japanese encephalitis virus from mosquitoes collected from mainland Australia. Am. J. Trop. Med. Hyg..

[26] Johnson B.J., Kerlin T., Hall-Mendelin S., van den Hurk A.F., Cortis G., Doggett S.L., Toi C., Fall K., McMahon J.L., Townsend M. (2015). Development and field evaluation of the sentinel mosquito arbovirus capture kit (SMACK). Parasit. Vectors.

[27] Boyle D.B., Dickerman R.W., Marshall I.D. (1983). Primary viraemia responses of herons to experimental infection with Murray Valley encephalitis, Kunjin and Japanese encephalitis viruses. Aust. J. Exp. Biol. Med. Sci..

[28] Mackenzie J.S., Johansen C.A., Ritchie S.A., van den Hurk A.F., Hall R.A. (2002). The emergence and spread of Japanese encephalitis virus in Australasia. Curr. Top. Microbiol. Immunol..

[29] Van den Hurk A.F., Smith C.S., Field H.E., Smith I.L., Northill J.A., Taylor C.T., Jansen C.C., Smith G.A., Mackenzie J.S. (2009). Transmission of Japanese encephalitis virus from the black flying fox, *Pteropus alecto*, to *Culex annulirostris* mosquitoes, despite the absence of detectable viremia. Am. J. Trop. Med. Hyg..

[30] Williams D.T., Daniels P.W., Lunt R.A., Wang L.-F., Newberry K.M., Mackenzie J.S. (2001). Experimental infections of pigs with Japanese encephalitis virus and closely related Australian flaviviruses. Am. J. Trop. Med. Hyg..

[31] Van den Hurk A.F., Jansen C.C., Loukas A. (2016). Arboviruses of Oceania. Neglected Tropical Diseases—Oceania.

[32] Van den Hurk A.F., Nisbet D.J., Hall R.A., Kay B.H., Mackenzie J.S., Ritchie S.A. (2003). Vector competence of Australian mosquitoes (Diptera: Culicidae) for Japanese encephalitis virus. J. Med. Entomol..

[33] Hemmerter S., Slapeta J., van den Hurk A.F., Cooper R.D., Whelan P.I., Russell R.C., Johansen C.A., Beebe N.W. (2007). A curious coincidence: Mosquito biodiversity and the limits of the Japanese encephalitis virus in Australasia. BMC Evol. Biol..

[34] Johnson P.H., Hall-Mendelin S., Whelan P.I., Frances S.P., Jansen C.C., Mackenzie D.O., Northill J.A., van den Hurk A.F. (2009). Vector competence of Australian *Culex gelidus* Theobald (Diptera: Culicidae) for endemic and exotic arboviruses. Aust. J. Entomol..

[35] Chapman H.F., Kay B.H., Ritchie S.A., van den Hurk A.F., Hughes J.M. (2000). Definition of species in the *Culex sitiens* subgroup (Diptera: Culicidae) from Papua New Guinea and Australia. J. Med. Entomol..

[36] Hall-Mendelin S., Jansen C.C., Cheah W.Y., Montgomery B.L., Hall R.A., Ritchie S.A., van den Hurk A.F. (2012). *Culex annulirostris* (Diptera: Culicidae) host feeding patterns and Japanese encephalitis virus ecology in northern Australia. J. Med. Entomol..

[37] Van den Hurk A.F., Johansen C.A., Zborowski P., Paru R., Foley P.N., Beebe N.W., Mackenzie J.S., Ritchie S.A. (2003). Mosquito host-feeding patterns and implications for Japanese encephalitis virus transmission in northern Australia and Papua New Guinea. Med. Vet. Entomol..

[38] Van den Hurk A.F., Smith I.L., Smith G.A. (2007). Development and evaluation of real-time polymerase chain reaction assays to identify mosquito (Diptera: Culicidae) blood meals originating from native Australian mammals. J. Med. Entomol..

[39] Van den Hurk A.F., Ritchie S.A., Johansen C.A., Mackenzie J.S., Smith G.A. (2008). Domestic pigs and Japanese encephalitis virus infection, Australia. Emerg. Infect. Dis..

[40] Bryan J.H., O’Donnell M.S., Berry G., Carvan T. (1992). Dispersal of adult female *Culex annulirostris* in Griffith, New South Wales, Australia: A further study. J. Am. Mosq. Control. Assoc..

[41] Van den Hurk A.F., Johnson P.H., Hall-Mendelin S., Northill J.A., Simmons R.J., Jansen C.C., Frances S.P., Smith G.A., Ritchie S.A. (2007). Expectoration of flaviviruses during sugar feeding by mosquitoes (Diptera: Culicidae). J. Med. Entomol..

[42] Hall-Mendelin S., Ritchie S.A., Johansen C.A., Zborowski P., Cortis G., Dandridge S., Hall R.A., van den Hurk A.F. (2010). Exploiting mosquito sugar feeding to detect mosquito-borne pathogens. Proc. Natl. Acad. Sci. USA.

[43] Ritchie S.A., Cortis G., Paton C., Townsend M., Shroyer D., Zborowski P., Hall-Mendelin S., van den Hurk A.F. (2013). A simple non-powered passive trap for the collection of mosquitoes for arbovirus surveillance. J. Med. Entomol..

[44] Huang B., Firth C., Watterson D., Allcock R., Colmant A.M., Hobson-Peters J., Kirkland P., Hewitson G., McMahon J., Hall-Mendelin S. (2016). Genetic characterization of archived Bunyaviruses and their potential for emergence in Australia. Emerg. Infect. Dis..

[45] Van den Hurk A.F., Hall-Mendelin S., Townsend M., Kurucz N., Edwards J., Ehlers G., Rodwell C., Moore F.A., McMahon J.L., Northill J.A. (2014). Applications of a sugar-based surveillance system to track arboviruses in wild mosquito populations. Vector Borne Zoonotic Dis..

[46] Ramirez A.L., Hall-Mendelin S., Doggett S.L., Hewitson G.R., McMahon J.L., Ritchie S.A., van den Hurk A.F. (2018). Mosquito excreta: A sample type with many potential applications for the investigation of Ross River virus and West Nile virus ecology. PLoS Negl. Trop. Dis..

[47] Fontaine A., Jiolle D., Moltini-Conclois I., Lequime S., Lambrechts L. (2016). Excretion of dengue virus RNA by *Aedes aegypti* allows non-destructive monitoring of viral dissemination in individual mosquitoes. Sci. Rep..

[48] Australian Technical Advisory Group on Immunisation (2018). Australian Immunisation Handbook.

[49] Australian Government Department of Health Japanese Encephalitis Virus Infection Case Definition—V1.1. http://www.health.gov.au/internet/main/publishing.nsf/Content/cda-surveil-nndss-casedefs-cd_je.htm.

[50] Mackenzie J.S., Broom A.K., Hall R.A., Johansen C.A., Lindsay M.D., Phillips D.A., Ritchie S.A., Russell R.C., Smith D.W. (1998). Arboviruses in the Australian region, 1990 to 1998. Comm. Dis. Intell..

[51] Mackenzie J.S., Gubler D.J., Petersen L.R. (2004). Emerging flaviviruses: The spread and resurgence of Japanese encephalitis, West Nile and dengue viruses. Nature Med..

[52] Mackenzie J.S., Lindsay M.D.A., Smith D.W., Imrie A. (2017). The ecology and epidemiology of Ross River and Murray Valley encephalitis viruses in Western Australia: Examples of One Health in action. Trans. R. Soc. Trop. Med. Hyg..

